# Distinctive physiological and molecular responses to cold stress among cold-tolerant and cold-sensitive *Pinus halepensis* seed sources

**DOI:** 10.1186/s12870-018-1464-5

**Published:** 2018-10-16

**Authors:** Khaled Taïbi, Antonio D. Del Campo, Alberto Vilagrosa, José María Bellés, M.P. López-Gresa, José M. López-Nicolás, José M. Mulet

**Affiliations:** 1 Faculty of Life and Natural Sciences, Ibn Khaldoun University, BP 78, Karman Campus, 14000 Tiaret, Algeria; 2 Re-ForeST, Research Institute of Water and Environmental Engineering (IIAMA), Universitat Politècnica de València, Camino de Vera s/n. 46022, Valencia, Spain; 3 Instituto de Biología Molecular y Celular de Plantas (IBMCP), Universitat Politècnica de València-Consejo Superior de Investigaciones Científicas, Camino de Vera s/n, 46022 Valencia, Spain; 4 Fundación Centro de Estudios Ambientales del Mediterráneo, Joint Research Unit University of Alicante – CEAM, University of Alicante, Alicante, Spain; 5 Departamento de Bioquímica y Biología Molecular-A, Facultad de Biología, Universidad de Murcia, Campus de Espinardo, 30100 Murcia, Spain

**Keywords:** *Pinus halepensis*, Climate change, Cold stress, Soluble sugars, Osmolytes, Antioxidants, Glutathione, Free amino acids, Seed source evaluation

## Abstract

**Background:**

Forest species ranges are confined by environmental limitations such as cold stress. The natural range shifts of pine forests due to climate change and proactive-assisted population migration may each be constrained by the ability of pine species to tolerate low temperatures, especially in northern latitudes or in high altitudes. The aim of this study is to characterize the response of cold-tolerant versus cold-sensitive *Pinus halepensis* (*P. halepensis*) seedlings at the physiological and the molecular level under controlled cold conditions to identify distinctive features which allow us to explain the phenotypic difference. With this objective gas-exchange and water potential was determined and the photosynthetic pigments, soluble sugars, glutathione and free amino acids content were measured in seedlings of different provenances under control and cold stress conditions.

**Results:**

Glucose and fructose content can be highlighted as a potential distinctive trait for cold-tolerant *P. halepensis* seedlings. At the amino acid level, there was a significant increase and accumulation of glutathione, proline, glutamic acid, histidine, arginine and tryptophan along with a significant decrease of glycine.

**Conclusion:**

Our results established that the main difference between cold-tolerant and cold-sensitive seedlings of *P. halepensis* is the ability to accumulate the antioxidant glutathione and osmolytes such as glucose and fructose, proline and arginine.

**Electronic supplementary material:**

The online version of this article (10.1186/s12870-018-1464-5) contains supplementary material, which is available to authorized users.

## Background

Climate change is driving dangerous and unpredictable conditions regarding temperature and water availability, which has a direct effect on plant species distribution [1]. For the Mediterranean region, the dry periods are predicted to become more severe than usual in many areas [2]. In addition, a high probability of very cold winters is expected in Europe as a result of sea ice cover decrease in the Barents-Kara Sea resulting from climate change [3]. Therefore, cold stress in several areas may constitute an important and recurring factor that will limit the survival of tree species mainly at higher latitudes or altitudes [4]. Consequently, long-lived species, such as forest trees, will need to be able to adapt to cold temperatures and endure off-season cold and freeze-thaw cycles that are becoming more frequent [5].

Trees at the seedling stage are physiologically and morphologically very sensitive to cold stress which can produce cell damage, reduce growth or even the tree’s chances of survival [6]. Therefore, the ability to cope with low temperatures is an important parameter driving the distribution of trees such as conifers in different locations [7].

At the molecular level cold stress alters membrane fluidity and consequently membrane permeability. Plants respond to cold stress by inducing physiological and molecular changes, including the plant metabolic profile. These changes may play an advantageous role in cell cryoprotection during cold stress or prior to freezing temperatures [8]. However, seedlings resistance to cold temperatures as well as the metabolic phenotype are likely to differ according to the plant genetic structure, the genetic origin or the provenance [9] and this difference may affect the plant’s ability to adapt to cold stress [10]. For instance differential sensitivity to winter cold was reported by Colombo et al. [11] in Douglas fir (*Pseudotsuga menziensii*) tissues from different seedling provenances. In a similar manner, provenance significantly affected stem and needle hardiness of Scotch pine (*Pinus sylvestris*) [7]. At the molecular level there are several descriptions of genes whose role is important in cold tolerance or acclimation [8] or even genes whose overexpression may increase cold tolerance [12], but there are no descriptions in the literature of metabolomic or molecular changes affecting different provenances of *P. halepensis* (Aleppo pine)*,* an important conifer species widely used for afforestation or reforestation programs under climates with dry, hot summers, and cold winters. This lack of information is limiting not only our basic knowledge on the species, but the chances of success in afforestation and reforestation programs. Selection and use of provenances and genotypes with potential resistance to sudden changes in environmental conditions, including cold, is crucial. But for many provenances, we do not have any information. There are several references in the literature studying the effect of cold in *P. halepensis* [13, 14], but none of them considers the effect of cold at the molecular or metabolic level. One advantage of having this information is that if we identify the physiological or molecular profile of cold-tolerant and cold-sensitive species, we can have a valuable tool to select provenances with more chances to survive under cold environments, as a higher content of a given metabolite may correlate with higher chances of survival.

In a previous study we compared the cold tolerance of various seed sources of *P. halepensis*, in a 4-year field experiment in which parameters such as tree diameter, height and survival were evaluated. This allowed us to identify cold-tolerant and cold-sensitive *P. halepensis* seed sources from a pool of different seed provenances [15, 16]. A similar strategy has been used previously to identify distinctive physiological and molecular traits relevant for drought stress [17]. In the present study, we have characterized the physiological and molecular responses of different *P. halepensis* seed sources under controlled cold stress conditions. This has allowed us to eliminate variables present in field studies, such as pathogens, different exposure or access to resources, wound stress caused by wind, rain or insect attack, etc. which can differentially affect plants and therefore influence the obtained results. The aim of this study is to characterize the molecular and physiological response of different *P. halepensis* seedlings under controlled cold stress conditions in order to identify distinctive traits between seed sources previously characterized in field as cold-tolerant or cold-sensitive [15].

## Results

In a previous study we characterized 11 different seed sources of *P. halepensis* out planted in a cold habitat and in a temperate habitat in a four-year field trial. Seed sources were selected considering not only spatial distribution but also environmental heterogeneity, prioritizing those that represent contrasted environments. The mentioned selection covered most of the climatic and ecologic regions of the natural range of this species in Spain with a wide spectrum of molecular and phenotypic variation, corresponding to nine Spanish provenances defined for the species and two seed orchards. We determined survival, plant height and plant stem diameter. Using this methodology we could characterize two seed sources as cold-tolerant (‘Lev’ and ‘Mst’) and two as cold-sensitive (‘Arg’ and ‘Bet’). A complete description can be found in the following references [15, 16]. For this study, the seeds were grown under greenhouse conditions in order to avoid the variability due to the field conditions.

### Physiological response

**Fig. 1:**
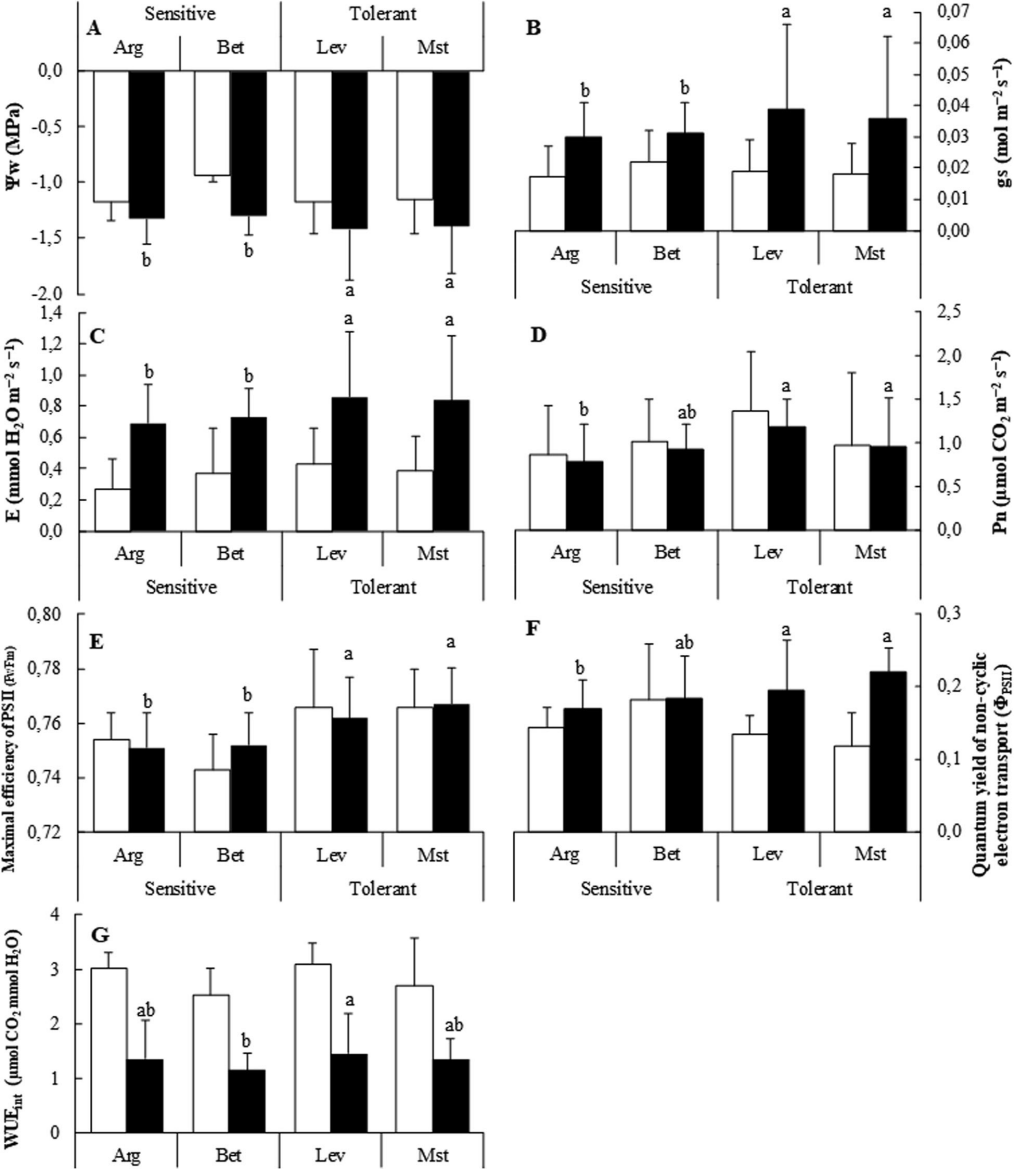
Physiological measurements of cold-sensitive and cold-tolerant *P. halepensis* seed sources. Water potential, gas exchange and photosynthesis measurements of cold-sensitive and cold-tolerant *P. halepensis* seed sources under control (white bars) and cold-stress (black bars) conditions. Water potential (Ψw) (**a**), stomatal conductance (gs) (**b**), transpiration (**e**) (**c**), Net photosynthesis (Pn) (**d**), Maximal efficiency of PSII (**e**), quantum yield of non-cyclic electron transport (ΦPSII) (**f**) and instantaneous water use efficiency (WUEinst) (**g**). The letters above the bars marks the significant difference among the cold-stressed seed sources following the post hoc Duncan’s test. Scale bars are mean + SE, being the number of samples *n* = 5

In order to attain the proposed objective, first we evaluated several physiological parameters both under control conditions and under cold stress (Fig. 1a-g). Under control growth conditions, we only observed minor differences among the tested seed sources. However, cold stress conditions generated several differences in the physiological and molecular responses of *P. halepensis* seedlings. Water potential (Ψ_w_) of the cold-tolerant seed sources exhibited a significant decrease under the cold stress, as compared to the cold-sensitive ones (*p*-value< 0.05) (Fig. 1a). In addition, cold-tolerant seed sources exhibited higher stomatal conductance (*gs*) (Fig. 1b), transpiration (*E*) (Fig. 1c) and net photosynthesis (*P*_*n*_) (Fig. 1d) (*p*-value< 0.05). Also, maximal efficiency of PSII (*F*v/*F*m), comprised between 0.74 and 0.78 for both treatments, was higher for cold-tolerant seedlings (p-value> 0.05) (Fig. 1e). Even though the quantum yield of non-cyclic electron transport (Φ_PSII_) was slightly higher in cold-sensitive seedlings under controlled conditions, cold-tolerant seedlings manifested higher values under cold stress (Fig. 1f). However, most of the differences, although statistically significant, were small.

**Fig. 2:**
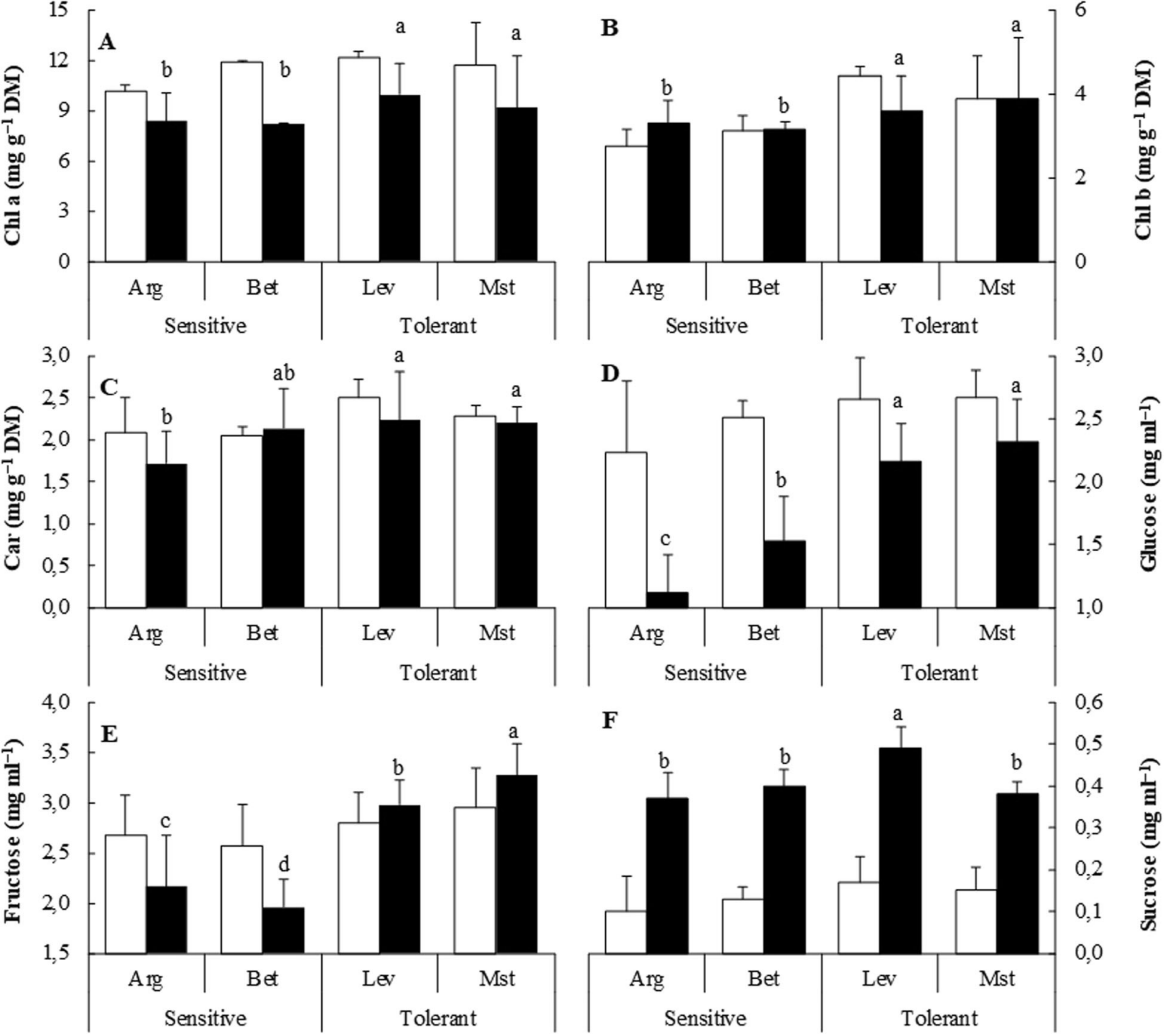
Photosynthetic pigments and soluble sugars content of cold-sensitive and cold-tolerant *P. halepensis* seed sources. Chlorophyll a (Chl a) (**a**), chlorophyll b (Chl b) (**b**), carotenoid (Car) (**c**), glucose (**d**), fructose (**e**) and sucrose (**f**) were determined for plants under control (white bars) and cold-stress (black bars) conditions. The letters above the bars marks the significant difference among the cold-stressed seed sources following the post hoc Duncan’s test. Scale bars are mean + SE, being the number of samples *n* = 5

We completed the physiological analysis by determining the concentration of the photosynthetic pigments. Chlorophyll a concentration was lower in all conditions for cold-tolerant seedlings (Fig. 2a), but concentrations of chlorophyll b and carotenoid remained similar for all seedlings under both conditions (Fig. 2b and c). Taken together, these data indicate that we could only observe minor changes in the investigated physiological parameters and in the content of photosynthetic pigments.

We also investigated the sugar content of the different seed sources and the changes under cold conditions. Cold stress induced a significant decrease in the glucose concentration. However, the glucose concentration of cold-tolerant seedlings was about two-fold higher than the concentration measured in cold-sensitive seedlings under cold stress conditions. Fructose concentrations were similar for all plants under control conditions, but cold-tolerant seedlings were able to increase fructose concentration under cold conditions, while the fructose concentration dropped about 20% in cold-sensitive provenances (Fig. 2d and e). Sucrose concentration increased upon cold stress, but we did not find important differences among cold-tolerant or cold-sensitive plants (Fig. 2f).

### Molecular response

**Fig. 3:**
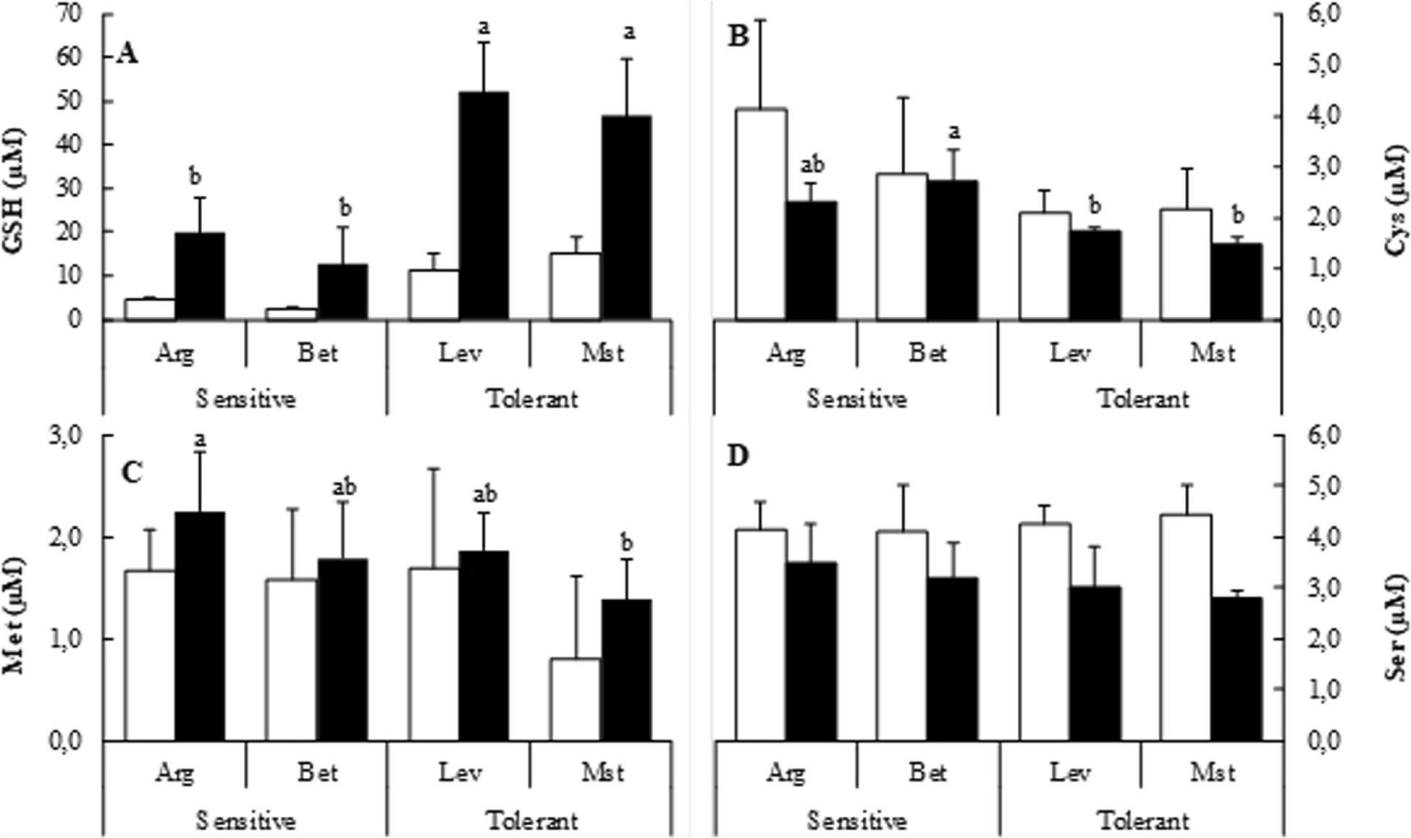
Glutathione, sulphur containing amino acids and serine content of cold-sensitive and cold-tolerant *P. halepensis* seed sources. Glutathione (GSH) (**a**), cysteine (Cys) (**b**), methionine (Met) (**c**) and serine (Ser) (**d**) were determined for plants under control (white bars) and cold-stress (black bars) conditions. The letters above the bars marks the significant difference among the cold-stressed seed sources following the post hoc Duncan’s test. Scale bars are mean + SE, being the number of samples *n* = 5

Cold stress is known to decrease membrane fluidity which can induce membrane damage and concomitantly water loss and oxidation, as the loss of fluidity affects the different electronic transport chains and increases the production of reactive oxygen species (ROS). Some amino acids can act as precursors for antioxidants or osmolytes or act themselves as osmolytes to prevent the deleterious effect of cold stress. Also, the tripeptide glutathione is a main determinant of the oxidative response in *P. halepensis* [17]. There is no description in the literature of the behaviour of the free amino acid pools under cold stress in the genus Pinus, so we investigated the glutathione and the complete free amino acid profiles of *P. halepensis* under the studied conditions, and the difference among provenances. The glutathione (GSH) concentration was higher in cold-tolerant seedlings, and also increased upon cold stress and accumulated approximately around five-fold more in cold-tolerant seedlings than in the cold-sensitive ones (Fig. 3a). Methionine (Met) concentrations increased under cold conditions while cysteine (Cys) and serine (Ser) concentrations decreased (Fig. 3b, d). However, there was no clear distinctive pattern between cold-tolerant and cold-sensitive seedlings for sulphur containing amino acids and serine, as the one observed for glutathione (Fig. 3).

**Fig. 4:**
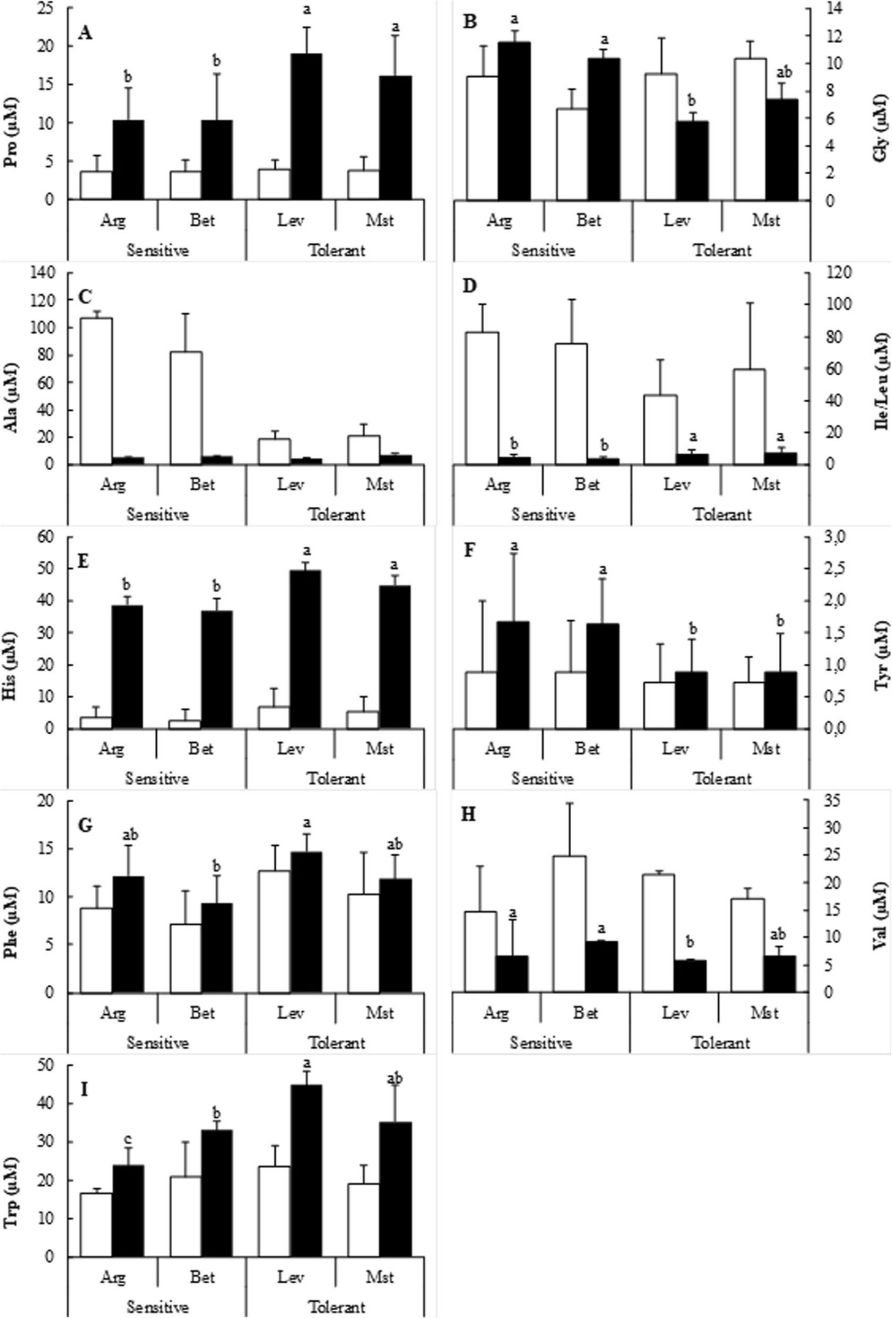
Non-polar amino acids and histidine content of cold-sensitive and cold-tolerant *P. halepensis* seed sources. Proline (Pro) (**a**), glycine (Gly) (**b**), alanine (Ala) (**c**), isoleucine/leucine (Ile/Leu) (**d**), histidine (His) (**e**), tyrosine (Tyr) (**f**), phenylalanine (Phe) (**g**), valine (Val) (**h**) and tryptophan (Trp) (**i**) were determined under control (white bars) and cold-stress (black bars) conditions The letters above the bars marks the significant difference among the cold-stressed seed sources following the post hoc Duncan’s test. Scale bars are mean + SE, being the number of samples *n* = 5

Proline (Pro) and glycine (Gly) are known to contribute to the osmotic adjustment and, as expected, proline accumulated significantly under cold stress and the accumulation in cold-tolerant seed sources was about two-fold higher (Fig. 4a). Interestingly, Gly concentrations were significantly higher in cold-tolerant seed sources under controlled conditions whereas, a significant increase was noted under cold stressed conditions in cold-sensitive seedlings, in contrast to a significant decrease in cold-tolerant seedlings (Fig. 4b). Gly is a component of the tripeptide GSH, so this decrease in cold-tolerant seedlings could be explained by the higher content of GSH observed in Fig. 3a.

Changes in the hydrophobic amino acids alanine (Ala), isoleucine/leucine (Ile/Leu) and valine (Val) were also noteworthy; they decreased dramatically under cold stress and were higher for cold-sensitive seedlings (Fig. 4c, d and h). The Ala concentration was five-fold higher in cold-sensitive seedlings (Fig. 4a). In contrast, the phenylalanine concentration (Phe) increased significantly under cold stress. Concentrations of Phe were significantly higher in cold-tolerant seedlings under both treatments (Fig. 4g).

For polar amino acids, histidine (His) concentrations increased about four-fold under cold stressed conditions and its concentration was higher in the cold-tolerant seedlings under both conditions (Fig. 4e). Under cold stress, seedlings accumulated higher concentrations of His and tyrosine (Tyr) (Fig. 4e and f). Tryptophan (Trp) concentrations did not differ among seedlings under controlled conditions. However, under cold stress, concentrations increased and the rise was significantly higher for cold-tolerant seedlings (Fig. 4i).

**Fig. 5:**
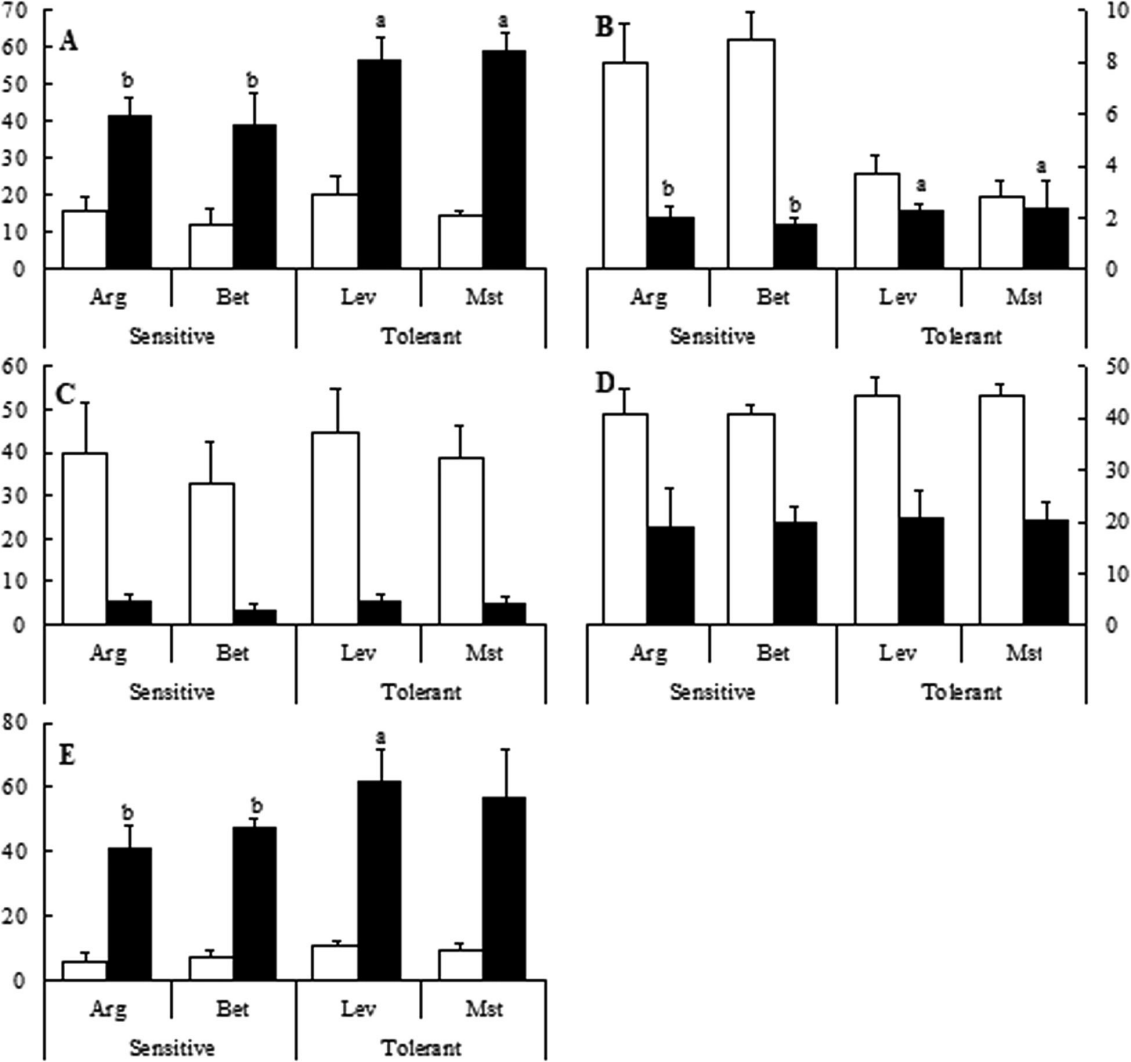
Polar or charged amino acids content of cold-sensitive and cold-tolerant *P. halepensis* seed sources. Arginine (Arg) (**a**), asparagine (Asn) (**b**), threonine (Thr) (**c**), lysine/glutamine (Lys) (**d**), glutamic acid (Glu) (**e**) and aspartic acid (Asp) (**f**) were determined under control (white bars) and cold-stress (black bars) conditions. The letters above the bars marks the significant difference among the cold-stressed seed sources following the post hoc Duncan’s test. Scale bars are mean + SE, being the number of samples *n* = 5

Regarding polar or charged amino acids, under cold stress there was an increase in the concentrations of arginine (Arg), glutamic acid (Glu) and aspartic acid (Asp), and in all cases the content under cold stress conditions was higher for cold-tolerant seed sources (Fig. 5a, e and f), while the situation was reversed for asparagine (Asn) (Fig. 5b). Cold stress induced a decrease in the concentration of Threonine (Thr) and lysine (Lys), but there were only minor differences among cold-tolerant and cold-sensitive seed sources (Fig. 5b-d).

## Discussion

*P. halepensis* is widely used in reforestation programs due to its aptitude to acclimate to different environmental and soil conditions [18]. In this study, we have compared at the physiological and molecular level the effect of cold stress among several *P. halepensis* seed sources, previously characterized in field experiments as cold-tolerant or cold-sensitive [15, 16]. The main objective of this work is to characterize the *P. halepensis* response to cold stress and identify distinctive physiological and molecular parameters among cold-tolerant and cold-sensitive provenances.

The main finding was that under control conditions all seed sources behave in a similar way, with very few exceptions. Most of the differences between cold-tolerant and cold-sensitive sources appeared when plants were under cold stress conditions. Although some differences were statistically significant, the physiological parameters evaluated in Fig. 1 and the photosynthetic pigments (Fig. 2a-c) presented small changes between different seed sources both in control and stress conditions, indicating that the parameters evaluated were behaving in a similar way in both provenances.

A different scenario appeared when we evaluated soluble sugars. These molecules have been related to stress tolerance in plants [19, 20]. Membrane fluidity iscompromised under cold stress and damage can induce water loss, ROS production and decreases intracellular vesicular transport which affects basic processes such as auxin transport and ion homeostasis [21]. Soluble sugars can act as osmoprotectants against membrane injuries and might be involved in ROS scavenging (hydroxyl radicals) under stress conditions [20, 22, 23]. Cold tolerant seedlings accumulate about 25% more glucose and 33% more fructose than cold-sensitive seedlings under stress conditions. Sucrose concentrations increased about four-fold upon cold stress, indicating that is involved in the cold response, but changes were similar in both provenances (Fig. 2d-f).

A side effect of the mentioned loss of membrane fluidity due to cold stress is the increase of oxidation due to the uncoupling of the electron transport chains. GSH is one of the most important thiols involved in the prevention of oxidative damage in plant cells [24, 25]. The results obtained throughout this study confirmed that GSH accumulation should be considered also as a distinctive and a key factor for cold tolerance in *P. halepensis.* We have shown that cold-tolerant seedlings exhibit a higher basal level under control conditions, and presented a four-fold increase under cold stress (Fig. 4a). Concomitantly cold-tolerant seedlings had less Cys and Ser (Figs. 3b, d and 4b). Cys is a constituent of the GSH tripeptide, which is biosynthesized from Ser, and its biosynthesis may become a limiting factor for stress tolerance [21].

Several studies have reported the change of the plant pattern of amino acids under cold stress [26]. However, this is the first complete analysis of free amino acids performed on different *P. halepensis* seed sources under cold stress. Free amino acid pools differ significantly under cold stress. In general, there were significant increases in the contents of Pro, His, Phe, Trp, Arg, Glu and Asp, along with a decrease in Ala, Iso/Leu, and Asn, concentrations (Figs. 4 and 5). Interestingly, Gly content increases significantly in cold-sensitive seedlings under cold-stress and decreases significantly in cold-tolerant ones (Fig. 4b). The variation of Gly content, similar to that of Cys, could be related to the GSH cell level, given that Gly is also a constituent of GSH [21].

It should be noted that the Trp levels increased under cold stress mainly for cold-tolerant seedlings. Similar results were reported before in *Picea mariana* seedlings [27] and *Pinus resinosa* needles [28]. In yeast, Trp uptake is very sensitive to membrane fluidity [29], so a similar process might occur in *P. halepensis*, as an increase in Trp content correlates with cold tolerance.

We have also determined that Pro and Arg concentrations increased upon cold stress. An increase in Pro concentration has also been observed in *Picea glauca* [27]*, Picea mariana* [28]*, Pinus resinosa* and *Picea obovata* [30]. Arg content increased also in response to cold stress in different woody species [31] . In our case, the increase in Pro and Arg concentrations were about two-fold higher and up to 60% in cold-tolerant seedlings respectively. This can be in part responsible for the in-field observed tolerance as Pro and Arg have an important role as osmolytes.

## Conclusion

The main conclusion of our study is that when we compare cold-tolerant and cold-sensitive seedling of *P. halepensis*, we could not detect marked changes in physiological parameters such as transpiration or photosynthesis rate, indicating that the distinctive response is not exerted at the physiological level. However when we investigated the metabolite concentrations we found that glucose, fructose, GSH, Pro, Arg, and Trp were higher in cold-tolerant seedlings. GSH is an antioxidant, while glucose, fructose, proline and arginine can function as osmolytes to prevent water loss. It is likely that Trp transport is impaired under cold conditions. So the increased accumulation of antioxidants and osmolytes, together with Trp, are the most distinctive features of cold-tolerant seedlings, indicating that the differential response is due to molecular rather than physiological changes.

An outcome of this report is that the analysis and determination of glucose, fructose, GSH, Pro, Arg and Trp might constitute a valuable tool in order to select previously uncharacterized provenances with higher chances to adapt to cold environments when data from long-term field trials are not available.

## Methods

### Experimental design and treatments

The study was performed on four *P. halepensis* seed sources selected among the recognized variability that have been tested before in field provenance trials over 4-years (1 year in the nursery+ 3 years in field) for survival, growth (height and stem diameterunder low temperatures [15, 16]. Based on these results, seed sources of ‘Maestrazgo Los Serranos’ (Mst) and ‘Levante Interior’ (Lev) were considered tolerant to low temperatures whereas those of ‘Bética Septentrional/Sur’ (Bet) and ‘Ibérico Aragonés’ (Arg) were considered sensitive. Seeds were grown in Forespot© 300 containers according to the common procedures reported for this species in the literature. The 16 cm deep plastic tray consists of 54 cells providing a density of 360 seedlings m^− 2^ [17, 31] filled with a mixture of sphagnum peat vermiculite-pine bark (3:1:1 *v*/v) and arranged in a complete random block design with six blocks where the different seed sources were randomized within the block. Seedlings were grown under controlled conditions in a growth chamber set at a 24 °C/16 °C day/night temperatures, photoperiod of 16 h (200 μmol m^− 2^ s^− 1^) and a relative humidity at 70% (Additional file 1: Figure S1). Irrigation was performed to full capacity every 2 days alternatively twice by water and once by complete Hoagland’s nutrient solution [32].

After a growth period of 25 weeks, healthy seedlings of similar size from each seed source, accounting for 15 replicates per seed source, were randomly assigned to control and low temperature treatment; control seedlings were sustained under the same growth conditions whereas low temperature treatment was applied throughout reducing temperature gradually, to avoid catastrophic xylem cavitation and deleterious associated effects, then set to a final temperature of 6 °C. Seedlings were kept under this temperature during 3 weeks and measurements were carried out at the end of the experiment.

### Physiological measurements

Seedling water potential (Ψ_w_, MPa) was measured with a Scholander-type pressure pump (model PMS-1000, PMS Instruments, Corvallis, OR, United States) on five seedlings selected randomly from each seed source per treatment. Instantaneous determinations of net CO_2_ assimilation (*P*_n_, μmol CO_2_ m^− 2^ s^− 1^), stomatal conductance (*g*_s_, mol m^− 2^ s^− 1^), transpiration *E* (mmol H_2_O m^− 2^ s^− 1^), and instantaneous water use efficiency (WUE_inst_; μmol CO_2_ mmol^− 1^ H_2_O) calculated as assimilation divided by transpiration *P*_n_/*E*, were also determined in five seedlings per seed source per treatment using a portable photosynthesis open-system (Model LI-6400, LI-COR Biosciences Inc., Lincoln, NE, United States). Gas exchange variables were estimated under conditions of saturating light (1500 μmol photon m^− 2^ s^− 1^), 25 °C and environmental CO_2_ (390 μmol mol^−1^CO_2_) maintaining the relative humidity in the chamber at approximately 55 ± 5%. All gas-exchange measurements were expressed as a function of needle-projected area. Maximal photochemical efficiency of PSII (*F*v*/F*m) was determined at predawn using a chlorophyll fluorometer (PAM 2000, Walz, Effedrich, Germany). Φ_PSII_ (quantum yield of non-cyclic electron transport) was estimated as (Fm′–Fs′)/Fm′ under steady-state conditions of illumination. It was determined early in the morning by using an open gas exchange system (LI-6400; LI-COR, Inc., Lincoln, NE, United States) with an integrated fluorescence chamber (LI-6400-40 leaf chamber fluorometer; LI-COR). Φ_PSII_ was determined in the same set of needles used for the gas exchange analysis. Maximal efficiency of PSII and ΦPSII were calculated according to Maxwell and Johnson [33].

### Molecular analysis

Chlorophyll a (Chl a), chlorophyll b (Chl b), and carotenoids (Car) concentrations were determined spectrophotometrically using the Lichtenthaler method [34]. Soluble sugars were determined by grinding 0.2 g of needles (fresh weight) in liquid nitrogen with a mortar and pestle, and then the homogenized powder was resuspended in 1 mL water and measured as described in Fayos et al. [35]. Specifically, the samples were incubated at 95 °C for 10 min, cooled on ice and centrifuged at 4 °C for 5 min to remove debris. The supernatants were filtered through Sep-Pak Plus C-18 solid phase cartridges (Waters). The soluble sugar fraction (mono and oligosaccharides) was separated by chromatography in a Waters 1525 HPLC system equipped with an evaporative light scattering detector (2424 ELSD). Aliquots (20 μl) were injected into the column ProntoSil 120-amino 3 μm (125 mm × 4.6 mm i.d.) with a Waters 717 autosampler. Elution was carried out at room temperature under isocratic conditions using a mixture of acetonitrile (J.T. Baker) and H_2_O (Milli-Q Millipore) (85:15) at a flow rate of 1 mL/min during 25 min. The conditions of the light scattering detector were the following: gain 75, data rate 1 pps, nebulizer heating 60%, drift tube 50 °C, and gas pressure 40 psi. Sugars were quantified with the Waters Empower software using glucose, fructose, sucrose, and sorbitol standards for the calibration curves. Glutathione (GSH) and free amino acids were extracted from 2 g of needles according to the method described in Mulet et al. [24]. In brief, plant material was pooled and homogenized in liquid nitrogen. Each pooled sample (0.10 g of FW) was heated 12 min at 95 °C in 2% isocitrate buffer (pH 2 with HCl). 1/10 dilutions of these extractions were injected in a Beckman Gold amino acid automatic analyser. The analysis was carried out following the protocol provided by the manufacturer, using a sodium citrate system and ninhydrin for detection.

### Statistical analysis

Data were subjected to analysis of variance with tolerance/sensitivity and seed sources as variables, seed source was nested to tolerance/sensitivity to determine differences among cold-tolerant and cold-sensitive seed sources separately, under controlled conditions, and then under cold stressed conditions. Additional analysis of variance was carried out to determine significant differences between means at *P* < 0.05 level. Homogeneous groups were separated using the Duncan’s test. In all cases, data were examined for normality and homogeneity of variances and assessed for any violations of assumptions using the Duncan’s test.

## Additional file

Additional file 1: **Figure S1.** Representative pictures of the greenhouse experimental set up. (JPG 613 kb)
