# Supplementary figures and images for: Distinctive physiological and molecular responses to cold stress among cold-tolerant and cold-sensitive Pinus halepensis seed sources
Source: BMC Plant Biol. 2018 Oct 16;18:236. doi: 10.1186/s12870-018-1464-5 (PMC6192292; doi:10.1186/s12870-018-1464-5)

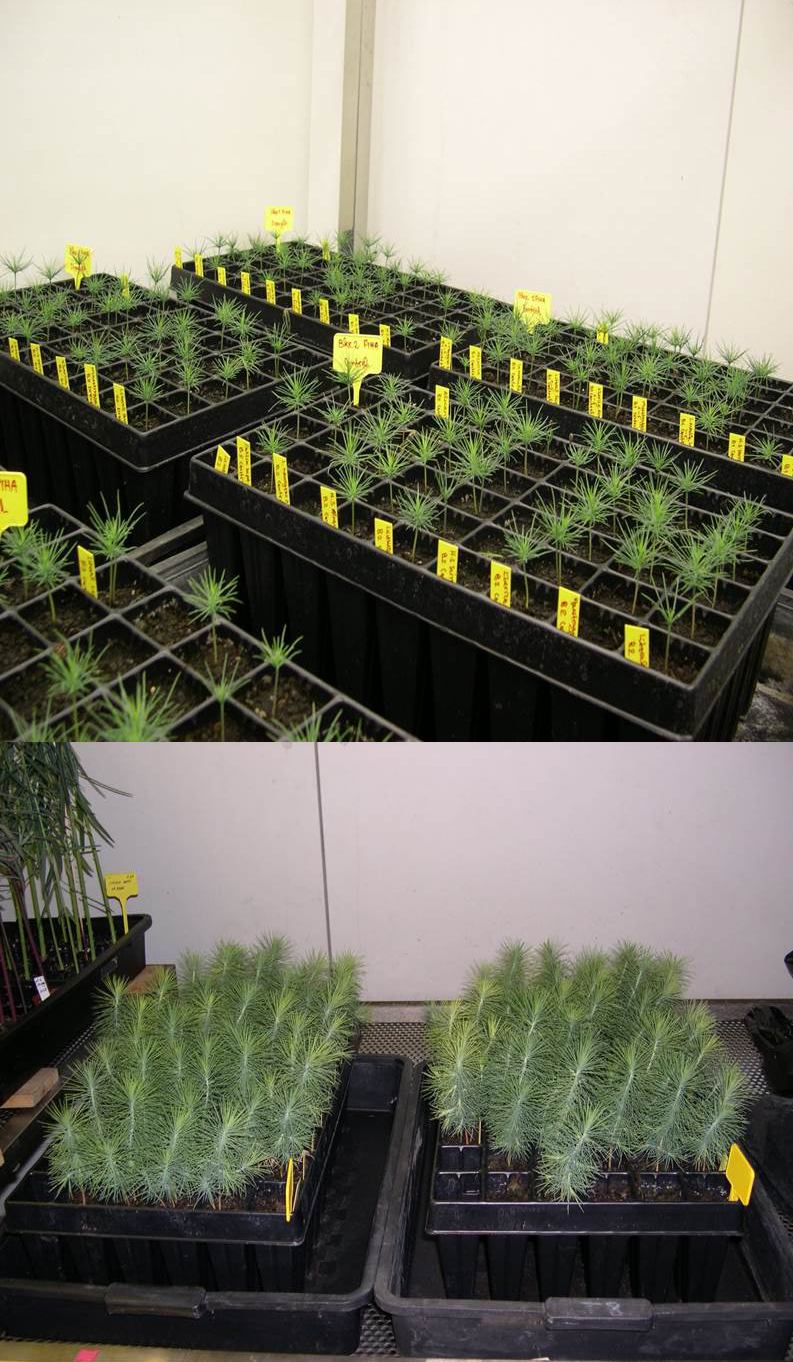

Supplement: Supplementary file 1 — Figure S1. Representative pictures of the greenhouse experimental set up. (JPG 613 kb) [file 12870_2018_1464_MOESM1_ESM.jpg]
